# p38 MAPK in cardioprotection – are we there yet?

**DOI:** 10.1111/bph.12901

**Published:** 2014-11-24

**Authors:** E D Martin, R Bassi, M S Marber

**Affiliations:** King's College London BHF Centre of Research Excellence, Cardiovascular Division, The Rayne Institute, St Thomas' HospitalLondon, UK

## Abstract

PKs transfer a phosphate from ATP to the side-chain hydroxyl group of a serine, threonine or tyrosine residue of a substrate protein. This in turn can alter that protein's function; modulating fundamental cellular processes including, metabolism, transcription, growth, division, differentiation, motility and survival. PKs are subdivided into families based on homology. One such group are the stress-activated kinases, which as the name suggests, are activated in response to cellular stresses such as toxins, cytokines, mechanical deformation and osmotic stress. Members include the p38 MAPK family, which is composed of α, β, γ and δ, isoforms which are encoded by separate genes. These kinases transduce extracellular signals and coordinate the cellular responses needed for adaptation and survival. However, in cardiovascular and other disease states, these same systems can trigger maladaptive responses that aggravate, rather than alleviate, the disease. This situation is analogous to adrenergic, angiotensin and aldosterone signalling in heart failure, where inhibition is beneficial despite the importance of these hormones to homeostasis. The question is whether similar benefits could accrue from p38 inhibition? In this review, we will discuss the structure and function of p38, the history of p38 inhibitors and their use in preclinical studies. Finally, we will summarize the results of recent cardiovascular clinical trials with p38 inhibitors.

## Tables of Links

**Table:** 

TARGETS	
**Other protein targets***^a^*	**Enzymes***^e^*
Cytokines	Glycogen synthase kinase
TNF-α	MKK3
**GPCRs***^b^*	MKK6
A2A receptors	MAPKAPK2
**Ion channels***^c^*	p38 MAPK
Cx43	PKA
**Transporters***^d^*	TAK1
SERCA2	

**Table:** 

LIGANDS	
ACh	NADPH
AEA	Nitric oxide (NO)
Angiotensin II	SB202190
ATP	SB203580
CD40 ligand	SCH58261
HU210	TxA2
LPS	U46619

These Tables list key protein targets and ligands in this article which are hyperlinked to corresponding entries in http://www.guidetopharmacology.org, the common portal for data from the IUPHAR/BPS Guide to PHARMACOLOGY (Pawson *et al*., 2014) and are permanently archived in the Concise Guide to PHARMACOLOGY 2013/14 (*^a,b,c,d,e^*Alexander *et al*., 2013a,b,c,d,e[Bibr b2],[Bibr b3],[Bibr b4],[Bibr b5],[Bibr b6]).

## p38 isoforms

The four isoforms of p38 are structurally homologous. The α and β isoforms share 74% sequence identity while the γ and δ isoforms are ∼70% similar to each other and ∼60% homologous to the α isoform. Significantly, the isoforms have different sensitivities to p38 inhibitors such as SB203580 (Eyers *et al*., 1998). These inhibitors compete with ATP and tend to inhibit p38α and p38β, over the p38γ and p38δ isoforms, because of small differences in side-chain length of their gatekeeper residues (Eyers *et al*., 1998). This feature can be used to create inhibitor resistant p38α and p38β knock-in mouse lines (O'Keefe *et al*., 2007). This strategy implicated p38α in both lethal myocardial ischaemic injury and cardioprotection following preconditioning (Kumphune *et al*., 2010; Sicard *et al*., 2010). Similarly, the p38α knockout mouse dies at embryonal day 10.5–11.5 (Adams *et al*., 2000; Allen *et al*., 2000) while ablation of the other isoforms, individually or p38δ and p38γ in combination, results in live mice without major abnormalities (Beardmore *et al*., 2005; Sabio *et al*., 2005). In addition, the loss of one isoform does not alter the expression or activity of the other isoforms. While p38α and p38β are ubiquitously expressed, the other isoforms possess a tissue-specific expression pattern, perhaps indicative of their different roles. p38γ has been implicated in myoblast differentiation and is enriched in skeletal muscle (Lechner *et al*., 1996). p38δ is predominantly expressed in lungs, kidney, testis, spleen, pancreas and small intestine (Kumar *et al*., 1997). The subcellular distribution of the isoforms may also indicate divergent roles, as p38α and p38β can be found in the cytosol and the nucleus while p38γ isoform is nuclear located in response to chronic pressure overload (Dingar *et al*., 2010). This theme can be further elaborated on by the substrates identified to be phosphorylated by the different isoforms. While most substrates such as myelin basic protein and activating transcription factor 2 are phosphorylated by multiple isoforms, some substrates appear to be uniquely phosphorylated by a particular isoform. The MAPK activated PK 2 (MAPKAPK2) has been show *in vitro* to be phosphorylated by α and β isoforms only (Cuenda *et al*., 1997). Identification of the repertoire of substrates of γ and δ has been more challenging because of the lack of a specific inhibitor for these isoforms. p38γ is unique in possessing a PDZ-binding domain sequence, KETXL, at its C-terminus likely responsible for the observed substrate bias towards α1 syntrophin, synapse-associated protein 90/ postsynaptic density 95 (Hasegawa *et al*., 1999; Sabio *et al*., 2004) and synapse-associated protein 97 (Sabio *et al*., 2005). The δ isoform has been shown to phosphorylate stathmin and Τau *in vitro* (Parker *et al*., 1998; Yoshida and Goedert, 2006) indicating the isoform is involved in regulating microtubule dynamics.

All four isoforms have been detected in the murine heart, but with higher levels of expression for p38α and p38γ compared with p38β and p38δ (Dingar *et al*., 2010). While the relationship between the isoforms is clearly complex and is still being elucidated, p38α appears most relevant to cardiovascular biology.

## Activation and structure of p38α MAPK

**Figure 1:**
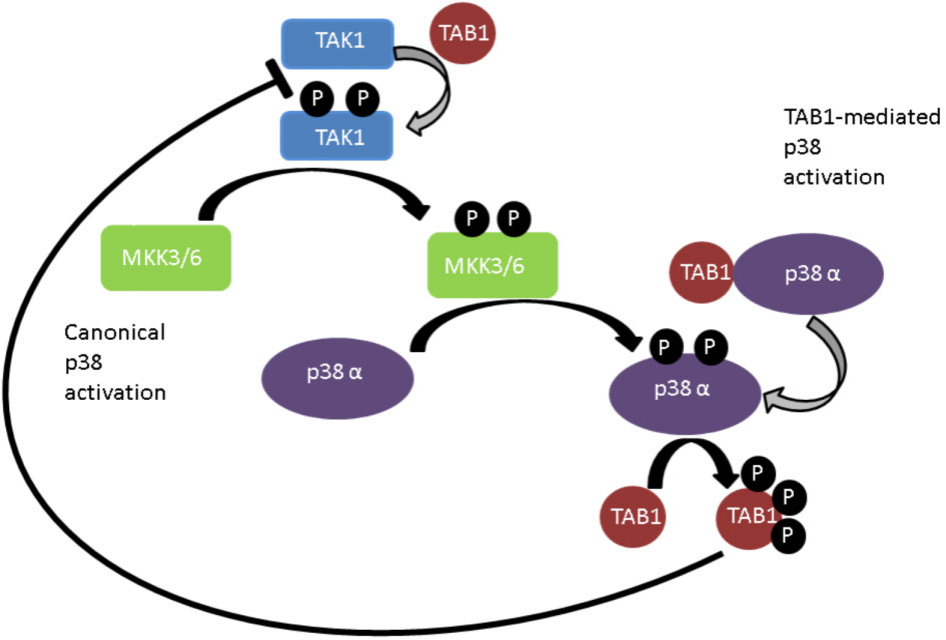
The major pathways of p38 activation. In the canonical activation pathway, TAK1 undergoes autophophorylation (grey arrow) upon binding to TAB1 (left portion of figure). This activated kinase in turn activates MKK3 or MKK6 by a transphosphorylation reaction (black arrow). Subsequently, the activated MKK phosphorylates p38α. p38α can also be activated by a more direct TAB1-mediated mechanism (right portion of figure). Binding of TAB1 to p38α induces autophosphorylation of p38α and activation of the kinase. TAB1 is also a substrate of p38α and phosphorylated TAB1 is a negative regulator of TAK1 autophosphorylation.

The structure of p38α is similar to that of other kinases with a smaller N-terminal lobe mainly composed of β-pleated sheets and a C-terminal lobe of α helices. The ATP-binding pocket/catalytic cleft is located at the juncture of these two lobes. In addition, an activation loop, with a number of conserved motifs, lies adjacent to the ATP-binding pocket. The activity of p38 is indicated by the dual phosphorylation of the TGY motif in the activation loop of the kinase (Taylor and Kornev, 2011). Upstream kinases, termed MAPK kinases (MKK), such as MKK3, MKK6, phosphorylate these residues (Figure 1) (Derijard *et al*., 1995; Raingeaud *et al*., 1996). The specific activator of the p38 isoform depends on the stimulus and the cell type (Remy *et al*., 2010). In turn, the MKKs are phosphorylated by upstream MAPK kinase kinases such as the TGF-β activated PK (TAK1). This hierarchical three-tiered level of activation is termed the canonical activation pathway. These components mediate the transduction of an environmental stress signal to elicit a response by the cell.

The conformation of the non-active kinase differs from that of the active kinase. In the inactive kinase, the activation loop occupies and sterically blocks the peptide-binding channel. The two lobes of the kinase are misaligned and therefore two residues, Lys^53^ of the N-terminal lobe and Asp^168^ of the C-terminal lobe, are not in close proximity. An active kinase requires cooperation between these key residues to bind the α phosphate group and ribose group of ATP and the companion Mg^2+^ ions (Wilson *et al*., 1996; Gum *et al*., 1998). Activation of the kinase via phosphorylation occurs in a highly ordered mechanism and results in the movement of the activation loop away from the peptide-binding channel (Humphreys *et al*., 2013). It is thought this movement exerts a ‘crank-handle effect’ on the tertiary structure of the kinase inducing the movement of the two lobes resulting in the alignment of Lys^53^ and Asp^168^ facilitating ATP binding. Overall these conformational changes result in the formation of the catalytic ‘hydrophobic spine’ of the kinase (Taylor and Kornev, 2011). In summary, the transphosphorylation of the TGY motif of the activation loop of the kinase, by upstream kinases, results in significant rearrangement of the kinase and dramatically increases its ability to function. Therefore, the activation of the kinase is a critical factor in the ability of a kinase to phosphorylate its substrates.

In addition to the canonical activation pathway, other additional activation mechanisms of p38 have been identified. Several groups, including ours, have reported a profound effect of binding of p38α isoform to the scaffold protein TAK1 binding protein 1 (TAB1) (Figure 1) (Ge *et al*., 2002; Tanno *et al*., 2003; Li *et al*., 2005). This binding results in activation of the kinase independent of upstream kinases through autophosphorylation. The binding of TAB1 to p38 resulted in significant alterations in the conformation of the kinase to facilitate active intramolecular phosphotransfer (De Nicola *et al*., 2013). This has been observed with the α isoform only (De Nicola *et al*., 2013). Interestingly, this scaffold-induced mode of activation was shown to be the mechanism through which p38 is activated during myocardial ischaemia (Kumphune *et al*., 2010). This indicates a significantly different mechanism than the more usual transactivation by MKK3/6 upstream kinases and may only occur under a limited number of circumstances.

## Importance of p38 in biology

There are numerous lines of evidence indicating the importance of p38 in biology.

The *Saccharomyces cerevisiae* homologue of p38α is the pheromone and stress-sensing *Hog1* gene (Bell *et al*., 2001). The high-osmolarity glycerol pathway has numerous functions in yeast including modulation of cell cycle progression at several stages and regulation of the biogenesis and export of mRNAs of stress-responsive genes (Duch *et al*., 2012; Regot *et al*., 2013). Similar to the mammalian system, *Hog1* is a component in a hierarchical activation cascade and is activated by phosphorylation of its activation loop motif by the upstream kinases, Pbs2 (Brewster *et al*., 1993). Another conserved feature is the mode of activation through autophosphorylation, in this case by binding to the scaffold protein, Ste5 (Bhattacharyya *et al*., 2006). The evolutionary conservation of the protein and its mechanisms of activation highlight its biological importance. In addition, the ubiquitous expression of the α isoform and studies indicating this is the predominant isoform mediating the harmful effects during myocardial infarction and the beneficial effects of preconditioning further underline its central function. This is further reflected in the significant interest in p38 MAPK attested by the high number (approaching 25 000) of papers returned on a PubMed search.

## Inhibitor design

Designing a specific p38 kinase inhibitor has proven to be challenging for a number of reasons. The 500 known mammalian PKs share a highly homologous ATP-binding site. Therefore, finding unique target areas for enabling the design of selective inhibitors has been difficult. For this reason, many kinase inhibitors have been shown to inhibit multiple kinase in addition to the original target (Bain *et al*., 2003). Nevertheless, the prominent role of p38α in cytokine production resulted in early success with inhibitors for use in arthritis, inflammatory bowel disease and chronic obstructive pulmonary disease (Lee *et al*., 1994). A number of crystal structures of p38α and p38γ in the presence or absence of a bound inhibitor are available in the protein databases and they provide a resource for future inhibitor design (Wilson *et al*., 1996; Wang *et al*., 1997; Bellon *et al*., 1999; Chang *et al*., 2002). The type I inhibitors binds in an ATP competitive manner. As discussed previously, the gatekeeper residue is a key determinant in the binding of the inhibitor and therefore the different isoforms have varied sensitivities to these types of inhibitors (Wilson *et al*., 1996; Gum *et al*., 1998). Members of this class also include SB 203580 and losmapimod. A challenge for efficient inhibition with type I inhibitors is that they must compete with physiological concentrations of ATP. To overcome these challenges, second types of inhibitors, which bind to the ATP-binding site and an adjacent region have been designed. Members of these type II inhibitors include BIRB 796 and VX745. BIRB 796 has been shown to inhibit all isoforms of the kinase (Kuma *et al*., 2005). These inhibitors make use of a pocket vacated by the DFG motif, which lies in the N-terminal portion of the activation loop. When the side chains of Asp and Phe are in the ‘out’ position, the kinase is inactive. BIRB796 binds to the ‘DFG out’ stabilizing it and preventing the kinase changing to an active conformation (Pargellis *et al*., 2002; Kuma *et al*., 2005). BIRB796 displays a slow onset of action as the DFG out conformer is rare. Despite the improved characteristics of type II inhibitors over type I inhibitors, translation to clinical studies has not achieved vastly superior results. Therefore, additional scaffolds and modes of inhibition have been sought. One such method has been termed type III inhibitors or allosteric kinase inhibitors. Exploiting less-conserved areas of the kinase distinct from the ATP-binding site offers the opportunity to develop unique inhibitors for a particular kinase with high selectivity because of the divergence of sequence in these less-conserved regions of the kinase.

Alternative sites for inhibitor binding distal to the ATP-binding site have been used by Murga and colleagues (Willemen *et al*., 2014). As discussed previously, they have shown that phosphorylation of the docking groove of p38 affects partner binding with reduced activation and of the kinase. A candidate inhibitor was designed to target this docking groove with some success. Targeting substrates that depend on this docking groove interaction would enable some modulation of p38 activity without global inhibition. While this approach of targeting p38 is attractive, the benefit would be increased if circumstance-specific inhibition could be achieved. Wang and colleagues have also adopted an alternative design strategy in targeting the TAB1-mediated autophosphorylation (Wang *et al*., 2013). A cell-permeable peptide was designed to inhibit p38 activity via the non-canonical autophosphorylation activation mechanism. We also had success with this strategy of disrupting p38α and TAB1 interaction, although the sequence targets were different (De Nicola *et al*., 2013). A detailed structural description of the binding of TAB1 to p38α was used to disrupt the interaction at key contact residues with a cell-permeable peptide preventing binding and therefore p38α autophosphorylation *in vitro* and *in vivo*. This strategy indicates that an inhibitor that could also disrupt this interaction would be advantageous in preventing p38α activation during myocardial ischaemia while maintaining its other cellular roles.

## Cardioprotection and p38

As summarized earlier, p38 is expressed ubiquitously and implicated in numerous and diverse pathologies. Therefore, perhaps not surprisingly, it plays an important role in many components of the cascade that leads from a healthy vasculature to myocardial infarction and heart failure. Rather than attempt to summarize these studies in the text, we have created a table (see Table 1) that highlights relevant publications addressing the role of p38 in this cascade from endothelial dysfunction → atherosclerosis → platelet activation/thrombosis → myocardial infarction → post-infarction remodelling, contractile dysfunction, arrhythmia and heart failure. As can be seen from the table, generally, p38 is activated by, and aggravates, cardiovascular pathologies. From an evolutionary standpoint, this seems counterintuitive as selection pressure has preserved p38 in yeast and man. However, the benefits of this kinase are apparent under the ‘ischaemic preconditioning’ heading within Table 1. Ischaemic preconditioning describes the paradoxical increase in resistance to lethal ischaemic injury that follows a brief, sublethal, period of ischaemia. Despite the fact that p38 is activated by, and contributes to, lethal ischaemic injury; it also is involved in triggering the protection of ischaemic preconditioning. Some authors have linked these effects; with transient p38 activation during short preconditioning cycles of ischaemia being responsible for diminished p38 activation during subsequent lethal more prolonged ischaemia (Nagarkatti and Sha'afi, 1998; Marais *et al*., 2001; Sanada *et al*., 2001) These opposing roles for p38 make it difficult to predict what may happen in a clinical trial of p38 inhibition.

**Table 1:** Summary of studies examining the role of p38 in the biological processes associated with cardiovascular disease and the effects of the use of p38 inhibitors on these processes

Biological process	Study	p38 manipulation	Observed effect
Endothelial dysfunction	Choi *et al*., 2014	Mouse mesenteric arteries and isolated endothelial cells, subjected to TNF-α in the presence of SB203580.	TNF-α-activated p38 and impaired endothelium-dependent relaxation to ACh; effect inhibited by SB203580.
	Kassan *et al*., 2014	Isolated coronary arterioles, mesenteric and femoral arteries from type 2 diabetic mice subjected to PEG-SOD and NADPH oxidase p22 (phox) siRNA.	Impaired endothelium-dependent relaxation and elevated p38 phosphorylation was reduced after treatment with ROS scavenger or by p22 (phox) down-regulation.
	Thakur *et al*., 2010	Mouse thoracic aortic ring preparation and cultured endothelial cells subjected to AngII, SCH58261 A_2A_ R antagonist and A_2A_R siRNA.	A_2A_R is involved in the regulation of endothelial ROS production by Nox2 and requires p38 MAPK signalling. Blockade or knockdown of A_2A_R inhibited p38 activation, inhibiting AngII effects on EC ROS production.
	Rajesh *et al*., 2010	Cultured primary HCAEC subjected to cannabinoid receptor agonists (AEA and HU210) in the presence of SB203580.	Inhibition of p38 by SB203580 attenuated AEA and HU210-induced cell death.
	Bao *et al*., 2007	Rat aortic ring preparations and MAPKAPK2 (−/−) mouse hearts subjected to treatment with AngII in the presence of SB239063.	AngII-induced hypertension and p38 phosphorylation was attenuated in MAPKAPK2 null mice. AngII-induced vascular dysfunction, superoxide anion production and cardiac remodelling were attenuated in aortic rings by SB239063.
	Riad *et al*., 2007	*In vivo* rat model of diabetes by administration of streptozotocin in the presence of SB239063.	SB239063 attenuated diabetes-induced p38 phosphorylation and improved impairments in LV and endothelial function.
	Widder *et al*., 2004	Rat aortic ring preparation of hearts subjected to ischaemia by LAD ligation in the presence of SB239063.	SB239063 preserved endothelium-dependent vasodilation, reduced vascular superoxide anion production and p38 and MAPKAPK2 phosphorylation.
	Ju *et al*., 2003	Spontaneously hypertensive rat and cultured HUVECs exposed to TNF-α and LPS in the presence of SB239063.	TNF-α and LPS-induced p38 activation and ICAM expression in HUVECSs and in aortas from hypertensive rats. All inhibited by SB239063 and endothelial dysfunction restored in aortic rings.
Atherosclerosis	Cheriyan *et al*., 2011	Randomized controlled trial of losmapimod (p38α/β MAPK inhibitor) on untreated hypercholesterolaemic patients. Endothelial function assessed by venous occlusion plethysmography.	Losmapimod attenuated inflammation by inhibiting p38 activity and improved NO-mediated vasodilation.
	Seeger *et al*., 2010	MicroCT and planimetry of mouse ApoE−/− aortas and isolated bone marrow-derived mononuclear cells.	Proangiogenic activation of p38 inhibited by SB and resulted in reduction of atherosclerotic lesion size, inflammation and increased vasculogenic cells.
	Sun *et al*., 2009	Isolated cultured macrophages from ApoE−/−, Npc1−/− mice exposed to free cholesterol.	Lipid accumulation in atherosclerotic plaques activates p38 and its targets Ctsk, S100a8, MMP8 and MMP14 via TLR signalling.
	Proctor *et al*., 2008	Transgenic mice expressing inducible SMC-specific DN-p38α subjected to carotid injury and ectopic expression of DN-p38α in cultured rat SMC, in presence of SB202190 or siRNA.	Mice expressing DN-p38α resistant to carotid injury and reduced p38 activity.
			SB202190 or siRNA blocked PDGF-induced p38 activation and cell proliferation, in A10 SMC.
	Morris *et al*., 2008	*In vivo* MRI assessment of ApoE−/− mouse aortas with AngII and SB239063.	SB239063 inhibited p38 activity, inflammation in atherosclerotic plaques and phagocytic activity of macrophages and diminished aortic root lesion size.
Platelet activation	Alrehani *et al*., 2013	HEK293 cells overexpressing α_IIb_β_3_, and protein phosphate 1 knockdown by siRNA.	p38 negatively regulated PP1cα-mediated adhesion to immobilized fibrinogen and fibrin clot retraction, which were abrogated in the presence of p38 inhibitor, SB203580.
	Yacoub *et al*., 2010	Isolated human and CD40−/− mouse platelets treated with soluble CD40 ligand (sCD40L).	Pro-thrombotic inflammatory CD40L enhanced platelet activation and aggregation via Rac1 and p38. SB203580, prevented p38 phosphorylation and impaired the effects of sCD40L on platelet P-selectin expression and aggregation.
	Rauch *et al*., 2007	Human vascular smooth muscle cell migration in Boyden–Chamber assay by fibrinogen in the presence of SB203580.	Fibrinogen-induced migration and mediated the activation of p38 in an ICAM-1-dependent manner. SB203580 inhibited p38 activity and cell migration.
	Shen *et al*., 2007	Isolated human platelets activated by exposure to collagen in the presence of resveratrol.	Resveratrol inhibited collagen-induced platelet aggregation, p38 MAPK phosphorylation and thromboxane A_2_ formation.
	Vega-Ostertag *et al*., 2007	Pressure-perfused CD1 mouse aorta, isolated platelets and cultured endothelial cells exposed to human aPL.	aPL-induced thrombosis and EC activation *in vivo* and aPL-induced monocyte adherence to HUVEC *in vitro*, were all attenuated by SB203580.
	Brooks *et al*., 2007	Isolated adult horse platelets and leukocytes following exposure to LPS *in vivo* and *in vitro* respectively.	LPS infusion enhanced p38 phosphorylation and TxA_2_ production in platelets and leukocytes. SB203580 attenuated LPS-induced TxA_2_ release in platelets.
	Sakurai *et al*., 2004	p38α+/− mice subjected to FeCl_3_-induced carotid injury model of thrombus formation.	Time to thrombotic occlusion was prolonged in p38α+/− mice and U46619-induced aggregatory response in platelets was impaired with poor binding to fibrinogen.
MI	Gray *et al*., 2011	Administration of GlcNAc-coated SB239063 in rat hearts subjected to regional ischaemia-reperfusion.	GlcNAc-decorated particles loaded with the p38 inhibitor SB239063 reduced apoptotic events and infarct size and improved acute cardiac function.
	Kumphune *et al*., 2010	Drug-resistant-p38α mouse hearts subjected to global ischaemia in the presence of SB203580.	p38α is the dominant active isoform during myocardial ischaemia that contributes to infarction
	Sy *et al*., 2008	PCADK-mediated delivery of SB239063 in rat hearts subjected to regional ischaemia.	Inhibition of p38 by SB239063 improved cardiac function following MI.
	Kaiser *et al*., 2004	Transgenic mice expressing DN-p38 and DN-MKK6, subjected to regional ischaemia by LAD ligation and ectopic expression in cultured rat neonatal cardiac myocytes and transgenic mice.	p38 functions as a pro-death signalling effector in both cultured myocytes as well as the intact heart.
	Tanno *et al*., 2003	MKK3-knockout mouse hearts and H9c2 expressing drug-resistant-p38α subjected to global ischaemia-reperfusion or SI, respectively, in the presence of SB203580.	Absence of MKK3 had no significant effect on post-ischaemic infarction or p38 activation. p38 activation is SB203580-sensitive and TAB1-associated and contributes to myocardial injury.
	Otsu *et al*., 2003	Regional ischaemia-reperfusion by coronary occlusion in p38α−/+ mouse hearts.	Ischaemia-reperfusion induces activation of p38 leading to injury. Reduction of p38α expression results in protection.
	Martin *et al*., 2001	Ectopic expression of drug-resistant-p38α in cultured rat adult cardiac myocytes and H9c2 myoblasts, subjected to simulated ischaemia.	Cardioprotective effect of SB203580 is through specific inhibition of p38α
	Ma *et al*., 1999	Langendorff-perfused rabbit hearts subjected to global ischaemia-reperfusion in presence of SB203580.	Inhibition of p38 by SB203580 is cardioprotective. SB203580 decreased myocardial apoptosis by ischaemia-reperfusion injury and improved cardiac function. This effect is most evident when SB203580 exposure precedes reperfusion.
	MacKay *et al*., 1999	Cultured rat neonatal cardiac myocytes subjected to simulated ischaemia in the presence of SB203580.	Inhibition of p38 activation during SI by SB203580 and reduced activation of caspase-3, affording protection against cardiomyocyte apoptosis.
	Yada *et al*., 2004	CD-1 mice, regional myocardial IR with or without FR167653	Pretreatment with FR167653 before IR reduced inflammatory cytokine expression and infarct size.
LV remodelling	Koivisto *et al*., 2011	Ectopic co-expression of p38α or p38β with CA-MKK3 or MKK6 in cultured rat neonatal cardiac myocytes.	p38β increased mRNA levels of pro-hypertrophic BNP and ANF, whereas p38α augmented the expression of the pro-fibrotic genes, CTGF, bFGF and MMP9.
	Lau *et al*., 2007	14-3-3(+/−) mouse coronary artery occlusion in the presence of SB202190 and cultured adult mouse cardiac myocytes.	SB202190 inhibited p38 activation and increased survival in 14-3-3(+/−mice), which developed pathological ventricular remodelling with increased cardiomyocyte apoptosis post MI.
	Engel *et al*., 2006	Rat hearts subjected to regional ischaemia by LAD Ligation in the presence of FGF-2 and SB203580.	Administration of SB203580 and FGF-2, post infarction resulted in cardiomyocyte mitosis, reduction of scarring and rescued heart function.
	Tenhunen *et al*., 2006	Rat hearts subjected to LAD ligation and adenoviral co-transfection of p38α and MKK3b.	p38 activation reduced infarct size, improved ejection fraction, fractional shortening, decreased LV diameter and increased capillary density and reduced apoptosis and fibrosis.
	Ren *et al*., 2005	Transgenic mice expressing cardiac-specific DN-p38α, subjected to regional ischaemia by LAD ligation.	DN-p38α mice had increased ventricular systolic function 7 days post MI and reduced infarct size, cardiomyocye apoptosis and Bcl-X_L_ deamination.
	Nishida *et al*., 2004	Cardiac-specific p38α conditional KO mice subjected to TAC and cultured isolated neonatal cardiac myocytes.	p38α plays a critical role in cardiomyocyte survival in response to pressure with no effect on hypertrophic growth, despite down-regulation of p38α.
	See *et al*., 2004	Rat hearts subjected to regional ischaemia by LAD ligation in the presence of RWJ-67657.	RWJ-67657 treatment following myocardial ischaemia had beneficial effects on LV remodelling and dysfunction.
	Andrews *et al*., 2003	Ectopic expression of CA-MKK6 in cultured rat neonatal cardiac myocytes.	MKK6-mediated activation of p38 prolonged contractile calcium transient, reduced SERCA2 expression, resulting in increased diastolic [Ca^2+^]_i_ and enhanced NFAT activity.
	Braz *et al*., 2003	Transgenic mice expressing cardiac- specific DN mutants of p38α, MKK3 or MKK6, subjected to abdominal aortic banding or AngII, ISO or PE.	p38 signalling antagonizes the hypertrophic growth response of the adult heart through a dominant mechanism involving crosstalk with the calcineurin–NFAT signalling pathway.
Myocardial contractile dysfunction	Wang *et al*., 2012	Langendorff-perfused HHcy−/+ mouse hearts subjected to global ischaemia-reperfusion and cultured rat adult cardiac myocytes subjected to Hcy in the presence of SB203580.	Ischaemia-reperfusion resulted in activation of p38 and in impaired cardiac relaxation, contractile function and increased apoptosis that was markedly exaggerated in HHcy mice and cardiomyocytes. SB203580 prevented the Hcy-induced changes.
	Yin *et al*., 2008	Rat hearts subjected to ischaemia by LAD ligation in the presence of SB203580.	SB203580 suppressed myocardial fibrosis and LV remodelling, attenuated p38 activation and expression of TNF-α, α-SMA and collagen I.
	Vahebi *et al*., 2007	Isolated skinned cardiac muscle fibre bundles from transgenic mice expressing DN-p38α and CA-MKK6.	Activation of p38α directly depresses sarcomeric function by decreased phosphorylation of α-tropomyosin, which is reversed by overexpression of DN-p38α.
	Bellahcene *et al*., 2006	Langendorff-perfused MKK3-knockout mouse hearts and isolated cardiac myocytes subjected to TNF-α.	TNF-α induces contractile depression by activating p38 in the intact heart and in isolated cardiac myocytes in an MKK3-dependent and SB203580-sensitive manner.
Arrhythmia	Lu *et al*., 2013	Whole cell patch-clamp analysis on isolated rabbit PV cardiomyocytes, treated with collagen.	Collagen modulated PV electrical activity with changes in AP morphology, increased triggered and spontaneous activity. SB203580 ameliorated collagen-induced p38 phosphorylation and arrythmogenesis.
	De Jong *et al*., 2013	Cultured isolated rat neonatal cardiac myocytes, subjected to mechanical stretch.	Stretch induced p38 phosphorylation and hypertrophy-related changes including increased cell diameter, reinduction of the foetal gene programme and cell death.
	Surinkaew *et al*., 2013	Rat hearts subjected to LAD ligation in the presence of SB203580.	SB203580 decreased ischaemia-induced ventricular tachycardia/ventricular fibrillation incidence and heat shock protein 27 phosphorylation, and increased connexin 43 phosphorylation.
	Tang *et al*., 2011	Rat hearts subjected to LAD ligation, Langendorff perfusion and cultured, isolated adult ventricular myocytes in the presence of SB203580 or auranofin.	Expression of ventricular K(+) channels is redox regulated and impairment of the Trx system post-MI heart contributes to I(to) remodelling through sustained activation of apoptosis signal-regulating kinase-1-JNK-p38 signalling.
Ischaemic preconditioning	Sicard *et al*., 2010	Drug-resistant-p38α or p38β mouse hearts subjected to ischaemic preconditioning in the presence of SB203580.	SB203580 attenuated activity of p38 and its substrates and abolished infarct size reduction by IP in WT and drug-resistant-p38β hearts but not in drug-resistant-p38α hearts. p38α is necessary for ischaemic preconditioning.
	Schulz *et al*., 2003	*In vivo* pig model of ischaemic preconditioning by LAD ligation (regional ischaemia).	IP increases co-localization of p38 with Cx43 and preserves phosphorylation of Cx43 during ischaemia. Inhibition of p38MAPK by SB203580 attenuated IP-induced IS-reduction and led to dephosphorylation of Cx43 that correlates with the propagation of I/R injury.
	Sanada *et al*., 2001	*In vivo* canine model of ischaemic preconditioning by coronary occlusion (regional ischaemia).	p38 MAPK activation during IP mainly mediates the cardioprotection followed by HSP27 phosphorylation/translocation. SB203580 treatment during IP blunted the infarct size limitation by IP and attenuated phosphorylation/translocation of HSP27.
	Marais *et al*., 2001	Langendorff-perfused rat hearts subjected to global ischaemia- reperfusion and cultured rat neonatal cardiac myocytes	p38 was activated during preconditioning and attenuated during subsequent ischaemia. Non-preconditioned hearts had elevated p38 activation in comparison. p38 inhibition by SB203580 during ischaemia and reperfusion is cardioprotective.
	Saurin *et al*., 2000	Ectopic expression p38α or p38β isoforms in cultured rat neonatal cardiac myocytes subjected to simulated ischaemia in the presence of SB203580.	Inhibition of p38 during prolonged ischaemia reduced injury and contributed to preconditioning-induced cardioprotection.
			p38α and p38β differentially activated or deactivated respectively, during ischaemia.
	Nagarkatti *et al*., 1998	Simulated ischaemia in rat myoblast cell line H9c2.	Inhibition of p38 before the onset of SI blocks preconditioning, but is protective during prolonged ischaemia.
	Weinbrenner *et al*., 1997	Langendorff-perfused rabbit hearts subjected to global ischaemia-reperfusion in presence of SB203580	Inhibition of p38 activation abolished protection in preconditioned hearts and cardiomyocytes.
	Tong *et al*., 2000	Langendorff-perfused rat hearts, preconditioned with or without SB202190	Preconditioning induced uptake of glucose was abrogated by the presence of SB202190

AEA, anandamide; ANF, atrial natriuretic peptide; AP, action potentials; aPL, antiphospholipid antibodies; ApoE, apolipoprotein E; AngII, angiotensin II; bFGF, basic fibroblast growth factor; CTGF, connective tissue growth factor; Ctsk, cathepsin K; Cx43, gap junction protein connexin43; DN, dominant negative; FGF, fibroblast growth factor; GlcNAc, *N*-acetylglucosamine; H9c2, rat myoblast cell line; Hcy, homocysteine; HHcy, hyperhomocysteinaemia; ICAM, intercellular cell adhesion molecule; IP, ischaemic preconditioning; I/R, ischaemia/reperfusion; IS, infarct size; ISO, isoproterenol; LAD, left anterior descending coronary artery; LV, left ventricle; MAPKAPK2, MAPK activated PK 2; MI, myocardial infarction; MMP, matrix metalloproteinase; NADPH, nicotinamide adenine dinucleotide phosphate; NFAT, nuclear factor of activated T-cell; Npc1, Niemann–Pick disease type C1; PCADK, poly(cyclohexane-1,4-diyl acetone dimethylene ketal); PE, phenylephrine; PEG-SOD, polyethylene glycol superoxide dismutase; PV, pulmonary vein; ROS, reactive oxygen species; SB, SB203580; SERCA2, sarcoplasmic reticulum Ca2+ ATPase; SI, simulated ischaemia; SMA, smooth muscle actin; SMC, smooth muscle cell; TAC, transverse aortic constriction; TLR, Toll-like receptor; TxA_2_, thromboxane A2; WT, wild type.

The findings that p38 activation aggravates many components of atherothrombosis and myocardial infarction, have laid the foundation for recent and relevant clinical trial activity. The companies with agents under investigation include GlaxoSmithKline (losmapimod, various trials), ArrayBioPharma (ARRY-371797, NCT02057341) and Bristol-Myers Squibb (BMS-582949, NCT00570752), although the latter programme seems inactive. GlaxoSmithKline has the most active programme with a number of phase 1 trials suggesting a potential benefit in patients with early (Cheriyan *et al*., 2011) and late (Sarov-Blat *et al*., 2010; Elkhawad *et al*., 2012) atherosclerosis. We have summarized these trials previously (Martin *et al*., 2012), and here will concentrate on the recently published SOLSTICE trial (Newby *et al*., 2014). SOLSTICE was a GlaxoSmithKline-sponsored phase 2 study performed by the Duke Clinical Research Institute (NCT00910962) (Newby *et al*., 2014). This study enrolled 535 patients with non-ST elevation myocardial infarction who were randomized in a 3:3:2 ratio to oral losmapimod (two regimes, both of 7.5 mg twice daily with, or without, a 15 mg initial dose) or placebo. The trial medication was initiated within 18 h of hospital presentation and continued for 12 weeks. The first dose had to be administered at least 2 h before planned percutaneous coronary intervention (PCI). This design attempted to ensure p38 was inhibited during any procedure-related exacerbation of myocardial injury. The primary end points were of safety and efficacy. The safety end point was mainly designed to detect hepatotoxicity. The efficacy end points were high-sensitivity C-reactive protein (hsCRP) at 12 weeks and biomarker measures of extent of myocardial infarction.

In terms of safety, there was no statistically significant increase in liver enzymes, but alanine transaminase elevations three times above the upper limit of normal were more common in the losmapimod groups. In addition, there was a statistically significant, but very small (∼2 μmol·L^−1^), increase in serum creatinine at 12 weeks in the losmapimod groups. Study drug discontinuation was twice as frequent among patients receiving losmapimod than among those receiving placebo. This observation was of borderline significance and the cause was unknown. Serious adverse events were very similar across groups.

In terms of efficacy, the principal end point, hsCRP concentration at 12 weeks, did not differ between groups. However, by 12 weeks, CRP had returned to baseline, so a discernable difference was unlikely. However, at earlier timepoints, both hsCRP and IL-6 concentrations were significantly lower in the losmapimod groups. Biomarkers of necrosis (creatine kinase and troponin I) did not differ by treatment group. However, in an MRI substudy (92 patients with paired MRI scans) losmapimod was associated with significant improvements in ejection fraction and left ventricular end-diastolic and end-systolic volumes, and also a significant reduction in discharge brain natriuretic peptide in the main cohort. Collectively, these signals hint of a benefit with losmapimod and p38 inhibition in the setting of myocardial infarction despite the fact that SOLSTICE failed to meet its primary efficacy end points. Moreover, this suggestion of benefit was observed despite a losmapimod oral dosing regime that probably only achieved a maximum of 50% inhibition of p38 at the time of peak effect which occurred 4 h after oral dosing (Barbour *et al*., 2013). This time–concentration profile matched the timing of PCI in SOLSTICE, where approximately 60% of patients in losmapimod and placebo groups underwent PCI at a median of about 5 h after randomization.

The potential efficacy signal in SOLSTICE has encouraged a much larger phase 3 trial with clinical end points. LATITUDE-TIMI 60 (Losmapimod to inhibit p38 MAPK as a therapeutic target and modify outcomes after an acute coronary syndrome–thrombolysis in myocardial infarction 60; NCT02145468) will involve approximately 26 000 patients worldwide with myocardial infarction and have a study design similar to SOLSTICE, but without the 15 mg loading group (i.e. losmapimod 7.5 mg twice per day vs. placebo). The primary efficacy end point is a composite of major adverse cardiovascular events comprising cardiovascular death, reinfarction or recurrent myocardial ischaemia requiring urgent revascularization at 12 weeks. The main inclusion criterion is type 1 myocardial infarction (an atherosclerotic plaque rupture event) and will include presentation electrocardiograms with both ST and no ST elevation. Study medication will be continued for 12 weeks and follow-up for 24 weeks. It is anticipated that the study will be completed in December 2018.

## Interaction with other signalling pathways

The p38 MAPK pathway is integrated with multiple other signalling pathways at different levels. For example, p38α has been shown to phosphorylate glycogen synthase kinase 3 and the Wnt signalling pathways (Bikkavilli *et al*., 2008; Thornton *et al*., 2008). p38α is part of numerous feedback mechanisms. A recent paper has also indicated p38α may participate in multiprotein complex hypertrophy mediated by a PKA anchoring protein, in combination with MKK3. In addition, p38α is located within a number of feedback loops. These studies reflect p38α as a signalling nexus of multiple signalling pathways the specifics of which are continuing to be elucidated. The multiple pathways also illustrate the complexity in targeting a single PK. It is clear that multiple related pathways may be affected if a single protein is inhibited. Therefore, the ideal inhibition of a kinase requires selective, temporally discrete and circumstance-specific inhibition.

Global inhibition of p38α can lead to detrimental effects to the homeostasis of the organism. Pharmacological inhibition of p38 has been achieved, but the results of the preclinical trials, although encouraging, have not translated to clinical trials with a similar level of success. Therefore, a more selective targeted inhibition of the kinase that could ameliorate the detrimental consequence of activation of the kinase while maintaining the essential functions of the kinase would be highly desirable. The recent studies indicating the TAB1-induced activation of the kinase that mediates myocardial damage would be an eligible target for inhibition and that would allow the circumstance-specific mode of activation to be exploited.

## Abbreviations

hsCRP: high-sensitivity CRP
PCI: percutaneous coronary intervention
TAB1: TGF-β-activated binding protein 1
TAK1: TGF-β-activated kinase 1
